# Video-Counseling Intervention to Address HIV Care Engagement, Mental Health, and Substance Use Challenges: A Pilot Randomized Clinical Trial for Youth and Young Adults Living with HIV

**DOI:** 10.1089/tmr.2020.0014

**Published:** 2021-01-07

**Authors:** Parya Saberi, Caravella McCuistian, Emily Agnew, Angie R. Wootton, Dominique A. Legnitto Packard, Carol Dawson-Rose, Mallory O. Johnson, Valerie A. Gruber, Torsten B. Neilands

**Affiliations:** ^1^ Department of Medicine, Center for AIDS Prevention Studies, University of California, San Francisco, San Francisco, California, USA.; ^2^ Department of Psychiatry, University of California, San Francisco, San Francisco, California, USA.; ^3^ Department of Community Health Systems, School of Nursing, University of California, San Francisco, San Francisco, California, USA.

**Keywords:** engagement in care, mental health, substance use, telehealth, video-counseling, youth living with HIV

## Abstract

**Background:** Substance use and mental health are two barriers to engagement in care and antiretroviral therapy (ART) adherence among youth and young adults living with HIV (YLWH). The consequences of suboptimal adherence in YLWH are increased risk of HIV transmission and a future generation of immunodeficient adults with drug-resistant virus.

**Methods:** The Youth to Telehealth and Texting for Engagement in Care (Y2TEC) study was a pilot randomized crossover trial that examined the feasibility and acceptability of a novel video-counseling series and accompanying text messages aimed at mental health, substance use, and HIV care engagement for YLWH. The intervention consisted of twelve 20–30-min weekly video-counseling sessions focused on identifying and addressing barriers to HIV care, mental health, and substance use challenges. Participants completed quantitative surveys at baseline, 4 months, and 8 months. Feasibility and acceptability were evaluated using prespecified benchmarks.

**Results:** Fifty YLWH aged 18–29 years living in the San Francisco Bay Area were enrolled. Eighty-six percent and 75% of participants were retained at 4 and 8 months, respectively. A total of 455 (76%) video-counseling sessions were completed. In 82% of sessions, participants responded that they strongly agreed/agreed with this statement: “I felt heard, understood, and respected by the counselor.” In 81% of sessions, participants responded that they strongly agreed/agreed with this statement: “Overall, today's session was right for me.” At baseline, among participants reporting mental health challenges, only 10% noted having ever received mental health services, and among those who reported substance use challenges, ∼19% reported ever receiving substance use services. After 4 months of the Y2TEC intervention, participants reported slightly higher ART adherence and HIV knowledge, decreased depression and anxiety, and reduced stigma related to mental health and substance use.

**Conclusions:** The Y2TEC intervention using video-counseling and text messaging was feasible and acceptable for YLWH. ClinicalTrials.gov ID: NCT03681145

## Introduction

Adolescents and young adults carry a significant burden for HIV risk, with the majority of new HIV cases in 2018 occurring among individuals between 13 and 34 years of age.^1^ Youth and young adults living with HIV (YLWH) experience suboptimal rates of antiretroviral therapy (ART) adherence,^2^ decreased linkage, and retention in care,^3^ and, therefore, higher rates of virological failure.^4^ These factors highlight the disparities that YLWH experience across all aspects of the HIV care continuum from diagnosis to virological suppression.^3^

In addition, YLWH are impacted by a range of psychosocial factors that can influence their HIV treatment engagement and retention. For example, YLWH experience high rates of mental health symptoms^5^ and limited access to mental health treatment.^6^ Mental health symptoms such as depression can further exacerbate disparities related to HIV care retention and adherence.^7,8^ Substance use is also a barrier for HIV treatment adherence for YLWH.^9^ Moreover, YLWH may experience other barriers such as unstable housing, food insecurity, low socioeconomic status, and stigma that are often entangled with mental health and substance use challenges and can additionally negatively impact HIV clinical outcomes.^10^

Despite the adverse impact that mental health and substance use challenges have on HIV clinical outcomes, there remains a shortage of behavioral health services and evidence-based interventions tailored to YLWH.^5^ Although several interventions have been developed to address mental health, substance use, and other psychosocial factors to improve ART adherence for adults,^11,12^ few interventions take into account unique aspects of youth developmental phases, culture, and priorities such as technology use.^13^ Technology-based behavioral interventions have demonstrated promise for adults living with HIV.^14,15^ Given that youth often have a greater breadth of experience using technology than older adults,^16^ technology should also be leveraged to address the disparities that YLWH face related to HIV care.

The need for remote services and technology-based health interventions is more important than ever given the severe acute respiratory syndrome coronavirus 2 (SARS-CoV-2) pandemic. Owing to the requirement of social distancing and quarantining that has been mandated in most U.S. cities, there is a concern that substance use and mental health challenges may increase.^17^ In addition, regulatory changes are being implemented to enable previously delivered in-person services to occur over technological platforms.^18^

A video-counseling intervention tailored to YLWH to address HIV health disparities would address many of the above-noted challenges. As a result, the Youth to Telehealth and Texting for Engagement in Care (Y2TEC) intervention was developed to examine the provision of mental health, substance use, and HIV care engagement counseling to YLWH using brief weekly video-counseling sessions and text messaging. Here we report the feasibility and acceptability of Y2TEC.

## Methods

### Study overview

The Y2TEC study was a randomized pilot study aimed at examining the feasibility and acceptability of a 12-session video-counseling and text messaging intervention targeting engagement in HIV care, mental health, and substance use for YLWH of ages 18–29 years residing in the San Francisco Bay Area. The intervention was delivered to participants in two groups (intervention and waitlist control) through 12 weekly 20–30-min video-counseling sessions for 4 months. The study included a crossover design with the waitlist control arm receiving the intervention after 4 months. This study design allowed us to examine the feasibility and acceptability of participant retention for 4 months before the start of counseling sessions in the waitlist control arm and participant retention for 4 months after the completion of counseling sessions in the intervention arm.

The University of California, San Francisco (UCSF) Institutional Review Board approved this study. The study obtained a Certificate of Confidentiality from the National Institutes of Health to protect the privacy of potential and enrolled study participants. The full study protocol is published elsewhere.^13^

### Participants

Participants were recruited through flyers at health clinics, Facebook, and Grindr advertisements, recontacting participants from prior studies who had consented to be contacted for future research, and a peer referral method. Youth of ages 18–29 years living with HIV, residing in or receiving medical care in the San Francisco Bay Area, who were English speaking, willing and able to provide informed consent, and had access to a mobile telephone with text messaging capability were included. Those planning to move out of California in the following 8 months or those with evidence of severe cognitive impairment, active psychosis, or intoxication at the time of consent were excluded.

Individuals who met study criteria after a telephone screening were asked to present identification to verify date of birth and proof of HIV status (a letter of diagnosis, laboratory results, or HIV medication prescription) either by text messaging a photograph to the encrypted study telephone or by bringing these documents to an in-person visit. Participants were provided a total of up to US $310 for completing all study activities. Incentives were provided using a reloadable debit card called ClinCard. In addition, participants were entered into a raffle for 10 chances to win $25 gift cards each time they responded to a text message requesting to confirm their contact information or answered the session rating questions after each video-counseling session.

### Procedures

Eligible participants attended an in-person enrollment visit where they provided written consent, completed a medical information release form, and responded to a Qualtrics (Provo, UT, USA; version March 2017) baseline survey. Then staff randomized participants to the intervention or waitlist control conditions. Randomization was done using SAS (version 9.4) based on randomly permuted block sizes to ensure equally sized groups, and all study staff were blinded to the randomization order.

Participants randomized to the intervention condition received their first counseling session in person. Those randomized to the waitlist control group initiated the intervention after 4 months, at which time they received the intervention completely remotely (i.e., without having met the counselor in person). This was done to simultaneously evaluate the acceptability of a fully remote versus hybrid in-person/online session delivery. All study procedures thereafter for both conditions were completed remotely through video-counseling using Zoom (HIPAA-compliant videoconferencing platform), text messaging using Mosio (HIPAA-compliant text messaging software), email, or telephone.

*The Y2TEC Intervention* is a 12-session video-based counseling series provided by trained counselors (i.e., one social worker or one psychology fellow) supervised by one clinical psychologist and the study principal investigator. This intervention is based on the information-motivation-behavioral skills model^19–21^ and includes psychoeducation/health education, motivational interviewing, and problem-solving therapy.^*^ Our intervention protocol was designed so that in each session, the counselor focused on (1) providing HIV, substance use, and/or mental health information; (2) increasing motivation to actively improve HIV care, enhance mental health, and/or reduce substance use; and (3) developing behavioral skills needed for improving HIV care, enhancing mental health care, and/or decreasing substance use.

During a 4-month period, participants completed up to twelve 20–30-min weekly video-counseling sessions. The number and duration of sessions were based on formative research with YLWH and consultation with clinical psychologists on the team. Before participation, participants provided background survey data about their HIV care, mental health, and substance use (see Measures section). This information was used to determine the participant's level of acuity to tailor the number of core sessions related to these domains.^*^ Therefore, participants received three to six psychoeducation and motivation-building core sessions covering engagement in HIV care, mental health, and substance use challenges. Those with higher acuity received two foundational modules for each of the three topics rather than one, amounting to a maximum of six core sessions.

Each participant received an initial session to orient them to the intervention. After completing the appropriate number of core sessions (ranging from three to six), the remaining four to seven sessions consisted of “menu” topics, and a final session (described hereunder). These menu sessions covered one of the following topics: (1) HIV care (in-depth), (2) mental health (in-depth), (3) substance use (in-depth), (4) lifestyle health, (5) social support, (6) family of origin, (7) romantic and sexual relationships, (8) self-identity and disclosure, (9) subsistence needs (housing, money, and resources), and (10) education and vocation. On the rare occasion that a client was in crisis and unable to engage on one of these topics, a “wildcard” session was offered that focused on crisis response and safety planning. Menu sessions were completed in any order and repeated as necessary.

During each menu session, participants were asked to identify a barrier related to the topic and how it may be impacting their HIV care or overall health. After the barrier was identified, the counselor assisted the participants in motivation enhancement and problem solving related to addressing the barrier. The counselor also encouraged the participants to set a goal related to the barrier to complete before the following session. After completing the core and the menu sessions, the 12th final session included reviewing the topics discussed and goals achieved, and identifying and providing community referrals for unmet needs.

*Bidirectional text messages* from research staff were sent to participants, including two appointment reminders sent 24 h and 15 min before scheduled counseling sessions, session rescheduling, monthly check-ins during the 4-month waiting period, session ratings after each counseling session, and a weekly text message with free fun local activities to facilitate rapport building.^13^ In line with providing information and motivation, text messaging was also used to support the video-counseling sessions by sending participants text messages related to goals set during the prior session and requested resources.

### Data collection and study outcomes

Fidelity to intervention protocol was examined during weekly meetings of counselors with the clinical psychologist and study principal investigator. In addition, the counselor completed session summary forms after each session, which included questions related to session length, technical issues, session topics covered, goals set, a session content fidelity checklist (including questions to ascertain whether the focus area was identified, education/information was provided, motivation was enhanced, barriers were identified, and problem solving was initiated), and a narrative progress note.

The primary outcomes of the study were feasibility and acceptability of the Y2TEC intervention for YLWH. Benchmarks for assessing feasibility and acceptability were determined *a priori*.^13^ For acceptability, participants completed a 30-item exit survey after intervention completion, which included questions about the participants' overall rating of the study, satisfaction with study procedures, and perception of improved ART adherence, mental health, and substance use with study participation. Study staff also administered two-item session rating questions through text messaging after each weekly video-counseling session, asking whether the participant “felt heard, understood, and respected by the counselor” and whether the “session was right” for them.^22^ The *a priori* benchmark for acceptability based on the exit survey and two-item session rating was ≥80%.

For feasibility, recruitment and retention numbers were tracked as well as counselor ratings on audio and video quality during each counseling session.^13^ *A priori* feasibility benchmarks included ≥70% of planned recruitment (i.e., ≥56 participants), ≥80% retention at 4 months, and ≥60% retention at 8 months. The benchmark for mean number of disconnections per video-counseling session was 1, and the benchmark for audio and video quality was 7 out of 10 (with 10 being perfect audio and video quality). Additional study outcomes included behavioral and HIV clinical outcomes. Self-assessment surveys at baseline, 4 months, and 8 months collected demographic, substance use, mental health, and ART adherence.

### Measures

Participants completed surveys primarily for two purposes, determination of level of mental health, substance use, and HIV acuity and assessment of intervention outcome. The items used to determine a participant's level of acuity to tailor the core sessions for substance use included Alcohol Use Disorders Identification Test (AUDIT),^23^ Drug Abuse Screening Test (DAST),^24^ and the Alcohol, Smoking, and Substance Involvement Screening Test (ASSIST)^25^; items for mental health included Generalized Anxiety Disorder (GAD),^26^ Patient Health Questionnaire (PHQ-9),^27^ Post-Traumatic Stress Disorder (PTSD) Checklist (PCL-5)^28^; and items for HIV clinical outcomes included the HIV Treatment Knowledge Scale,^29^ a self-reported detectable HIV viral load, <80% self-reported ART adherence, or no report of appointments with a health care provider (HCP) in the past 6 months and no upcoming appointments scheduled.

AUDIT consists of 10 questions summed to give an alcohol dependence score: “Low” (0–7), “Moderate” (8–15), “High” (16–19), and “Dependence” (20–40). DAST sums questions to scale the results as “No” (0), “Low” (1–2), “Moderate” (3–5), “Substantial” (6–8), and “Severe” (9–10) drug use. A score of ≥8 on AUDIT, ≥3 on DAST, monthly or more frequent drug use (besides marijuana or tobacco) on ASSIST, or daily use of marijuana or tobacco on ASSIST was used to identify the need for two core substance use sessions. GAD scores questions such that a high score (10–21) indicates evidence of anxiety and a score <10 indicates minimal–mild anxiety. PHQ-9 scores depression-related questions that are categorized as “None” (0), “Minimal” (1–4), “Mild” (5–9), “Moderate” (10–14), “Moderately severe” (15–19), and “Severe” (20–100). PCL-5 groups the questions as either unlikely to have PTSD (0–32) or high likelihood of having PTSD (33–80). A score of ≥10 on GAD, ≥10 on PHQ-9, or ≥33 on PCL-5 resulted in two mental health sessions. The HIV treatment knowledge scale asks a series of questions about the participant's knowledge and scores the results as “Inadequate” (0–11) and “Adequate” (12–15) HIV treatment knowledge. HIV treatment knowledge ≤12 (equating to approximately >20% incorrect responses), a detectable HIV viral load, not taking ART, or not having had a provider appointment in the past 6 months and not having a scheduled upcoming appointment resulted in two HIV core sessions.

Demographic information as well as other items related to HIV, substance use, and mental health. Additional items were used to explore perceived HIV stigma,^30^ substance use, and mental health stigma (Substance Abuse and Mental Health Services Administration [SAMHSA] stigma assessments^31^), social isolation (Patient-Reported Outcomes Measurement Information System [PROMIS]^32^), sleep quality (Pittsburgh Sleep Quality Index [PSQI]^33^), Adverse Childhood Experiences Score (ACES),^34,35^ resilience,^36,37^ HCP engagement,^38^ and self-reported ART medication adherence.^39^

For HIV stigma (0–16), SAMHSA stigma (1–24), PROMIS (14–70), ACES (0–10), and HCP engagement (0–52), higher scores indicate greater HIV stigma; stigma about mental health, alcohol, and drug use; social isolation; more adverse childhood events; and worse engagement with HCPs, respectively. In the PSQI (0–20), scores of ≥5 indicate poor sleep quality. The brief resilience scale takes a mean score of the items, with a high score indicative of resilience and a low score suggesting vulnerability to stress. Unmet subsistence needs (defined as difficulty gaining access to a bathroom, place to wash, clothing, food, or a place to sleep) were also assessed.^40^ The self-report medication adherence gives a mean score (0–100) of the three items on adherence, with high scores showing better reported adherence.

### Data analysis

For the baseline, 4-month, and 8-month assessment surveys, we used descriptive statistics to generate frequencies for categorical variables and means and standard deviations (SDs) for continuous variables. In line with recommendations in the research methods literature and from NIH, inferential analyses were not performed on these pilot study data.^41,42^ All analyses were conducted using Stata, version 15 (StataCorp, College Station, TX).

## Results

### Demographics

From August 15, 2018 to April 2, 2019, we recruited 50 participants who had a mean age of 25.3 years, were 82% cisgender men, 44% Latino, 16% black/African American, and 74% gay identified (Table 1). Approximately 58% had completed secondary education. Despite 68% reporting part-time or full-time work, 54% indicated financial hardship, and 38% reported ever being homeless.

**Table 1.** Characteristics of Participants by Study Arm

	Intervention	Waitlist control
	N = 25	N = 25
Age, mean (SD)	25.8 (2.7)	24.7 (3.2)
Race, *N* (%)		
Latino	10 (40.0)	12 (48.0)
Black or African American (non-Latino)	5 (20.0)	3 (12.0)
White (non-Latino)	5 (20.0)	2 (8.0)
Asian or Pacific Islander (non-Latino)	3 (12.0)	4 (16.0)
Other (non-Latino)	2 (8.0)	4 (16.0)
Gender, *N* (%)		
Male	22 (88.0)	19 (76.0)
Female	3 (12.0)	3 (12.0)
Other	0 (0.0)	3 (12.0)
Sexual orientation, *N* (%)		
Heterosexual/straight	3 (12.0)	3 (12.0)
Gay	19 (76.0)	18 (72.0)
Other	3 (12.0)	4 (16.0)
Income, *N* (%)		
<$9999	9 (36.0)	9 (36.0)
$10,000–29,999	8 (32.0)	6 (24.0)
≥$30,000	8 (32.0)	8 (32.0)
Decline to answer	0 (0.0)	2 (8.0)
Financial situation, *N* (%)		
I have enough money	10 (40.0)	11 (44.0)
Barely/cannot get by	14 (56.0)	13 (52.0)
Decline to answer	1 (4.0)	1 (4.0)
Education, *N* (%)		
High school or less	10 (40.0)	11 (44.0)
More than high school	15 (60.0)	14 (56.0)
In school, *N* (%)		
In school	9 (36.0)	9 (36.0)
Not in school	16 (64.0)	16 (64.0)
Working, *N* (%)		
Working (full time or part time)	17 (68.0)	17 (68.0)
Not working	8 (32.0)	8 (32.0)
Living situation, *N* (%)		
Your own house/apartment/room	8 (32.0)	11 (44.0)
Parent's house/apartment	4 (16.0)	5 (20.0)
Other	13 (52.0)	9 (36.0)
Ever homeless, *N* (%)		
No	17 (68.0)	13 (52.0)
Yes	8 (32.0)	11 (44.0)
Decline to answer	0 (0.0)	1 (4.0)
Ever jail, *N* (%)		
No	20 (83.3)	20 (83.3)
Yes	4 (16.7)	4 (16.7)
Decline to answer	1 (4.0)	1 (4.0)
Unmet subsistence needs, *N* (%)		
Difficulty finding enough to eat	6 (24.0)	4 (16.0)
Difficulty finding place to wash	3 (12.0)	2 (8.0)
Difficulty finding clothing	3 (12.0)	1 (4.0)
Difficulty finding place to sleep	1 (4.0)	2 (8.0)
Difficulty finding a bathroom	1 (4.0)	2 (8.0)
No. of unmet subsistence needs, *N* (%)		
0	18 (72.0)	20 (80.0)
1	4 (16.0)	3 (12.0)
≥2	3 (12.0)	2 (8.0)

### Feasibility

From a total of 68 eligible individuals, 50 (74%) consented to participate. The main reasons for not consenting were loss to follow-up. Among the 25 participants in the intervention arm, 23 (92%) completed the intervention at 4 months, and 17 (74%) completed the 8-month assessment. From the 25 participants in the waitlist control arm, 20 (80%) completed the 4-month assessment and 17 (85%) completed the intervention at 8 months. Main reasons for lack of study completion were loss to follow-up. A total of 455 video-counseling sessions were completed out of 600 possible sessions (76%), with 263 sessions in the intervention arm and 192 sessions in the waitlist control arm. Among the completed video-counseling sessions, 9% were initial, 37% core, 45% menu, 1% wildcard, and 8% final sessions. Mean session length was 26 min (range = 11–40 min). The majority of sessions were completed at the participant's home (53%), workplace (12%), or in a car (11%). There was a mean of 1.7 disconnections per session. The audio and video quality of the sessions (as rated by participants) were 8.5 and 8.3 out of 10, respectively.

### Acceptability

Overall, 93% of participants reported their experience with the intervention as excellent to very good (Table 2). Approximately 95% of participants reported being extremely to very satisfied with the remote nature of the intervention. About 98% were extremely to very satisfied with using video-counseling for sessions with a counselor and 88% extremely to very satisfied with the content of the sessions. Nearly 88% of participants noted being extremely to very satisfied with meeting the counselor remotely (rather than in person). Over 91% participants in the intervention arm and over 94% of those in the waitlist control arm were extremely to very comfortable speaking to a counselor that they had only met once in person or not met in person, respectively. Approximately 90% reported the video-counseling platform to be extremely to very easy to use, and 88% reported that they were extremely to very likely to recommend the study to a friend.

**Table 2.** Acceptability by Study Arm

	Intervention	Waitlist control
	N = 23	N = 17
How would you rate your overall experience with the Y2TEC study? *N* (%)		
Excellent–very good	20 (87.0)	17 (100.0)
Good–very poor	3 (13.0)	0 (0.0)
How satisfied were you with participating in a research project where most study activities were conducted remotely? *N* (%)		
Extremely–very satisfied	21 (91.3)	17 (100.0)
Somewhat satisfied–extremely unsatisfied	1 (4.3)	0 (0.0)
Decline to answer	1 (4.3)	0 (0.0)
How satisfied were you with participating in a study where you used a video-counseling platform for sessions with a counselor? *N* (%)		
Extremely–very satisfied	22 (95.7)	17 (100.0)
Somewhat satisfied–extremely unsatisfied	1 (4.3)	0 (0.0)
How satisfied were you with the need to meet with the counselor weekly for video-counseling sessions? *N* (%)		
Extremely–very satisfied	20 (87.0)	16 (94.1)
Somewhat satisfied–extremely unsatisfied	3 (13.0)	1 (5.9)
How satisfied were you with the content of the video-counseling sessions? *N* (%)		
Extremely–very satisfied	19 (82.6)	16 (94.1)
Somewhat satisfied–extremely unsatisfied	3 (13.0)	1 (5.9)
Decline to answer	1 (4.3)	0 (0.0)
How satisfied were you with the need to meet remotely with the counselor rather than in person? *N* (%)		
Extremely–very satisfied	19 (82.6)	16 (94.1)
Somewhat satisfied–extremely unsatisfied	2 (8.7)	1 (5.9)
Decline to answer	2 (8.7)	0 (0.0)
How satisfied were you with the security and privacy of the video-counseling sessions? *N* (%)		
Extremely–very satisfied	19 (82.6)	14 (82.4)
Somewhat satisfied–extremely unsatisfied	2 (8.7)	3 (17.7)
Decline to answer	2 (8.7)	0 (0.0)
How satisfied were you with the text messages from the study staff? *N* (%)		
Extremely–very satisfied	12 (52.5)	15 (88.2)
Somewhat satisfied–extremely unsatisfied	9 (39.1)	2 (11.8)
Decline to answer	2 (8.7)	0 (0.0)
How satisfied were you with the use of the ClinCard to receive your study incentives? *N* (%)		
Extremely–very satisfied	19 (82.6)	16 (94.1)
Somewhat satisfied–extremely unsatisfied	2 (8.7)	1 (5.9)
Decline to answer	2 (8.7)	0 (0.0)
Did you meet with the counselor in person before beginning your video-counseling sessions? *N* (%)		
Yes	23 (100.0)	0 (0.0)
No	0 (0.0)	17 (100.0)
Rate your comfort level in speaking to a counselor that you only met once,^a^*N* (%)		
Extremely–very comfortable	21 (91.3)	0 (0.0)
Somewhat comfortable–extremely uncomfortable	1 (4.8)	0 (0.0)
Decline to answer	1 (4.8)	0 (0.0)
Rate your comfort level in speaking to a counselor that you did not meet in person,^b^*N* (%)		
Extremely–very comfortable	0 (0.0)	16 (94.1)
Somewhat comfortable–extremely uncomfortable	0 (0.0)	1 (16.7)
How easy or difficult was it to use your personal cell phone, computer, or tablet for video-counseling sessions? *N* (%)		
Extremely–very easy	20 (87.0)	17 (100.0)
Somewhat easy–extremely difficult	3 (13.0)	0 (0.0)
How easy or difficult was it to use the video-counseling platform to meet with the counselor? *N* (%)		
Extremely–very easy	19 (82.6)	17 (100.0)
Somewhat easy–extremely difficult	3 (13.0)	0 (0.0)
Decline to answer	1 (4.3)	0 (0.0)
How easy or difficult was it to hear the counselor in the video-counseling sessions? *N* (%)		
Extremely–very easy	14 (60.9)	16 (94.1)
Somewhat easy–extremely difficult	8 (34.8)	1 (5.9)
Decline to answer	1 (4.3)	0 (0.0)
How easy or difficult was it to see the counselor in the video-counseling sessions? *N* (%)		
Extremely–very easy	18 (78.3)	16 (94.1)
Somewhat easy–extremely difficult	4 (17.4)	1 (5.9)
Decline to answer	1 (4.3)	0 (0.0)
Did you ever have trouble with internet access or Wi-Fi during your video-counseling session? *N* (%)		
Yes	12 (52.2)	4 (23.5)
No	11 (47.8)	13 (76.5)
Did you ever have trouble finding a private place to have your video-counseling session that felt confidential? *N* (%)		
Yes	4 (17.4)	5 (29.4)
No	19 (82.6)	12 (70.6)
Did you ever receive help from anyone to use the video-counseling platform for your sessions? *N* (%)		
Yes	3 (13.0)	2 (11.8)
No	19 (82.6)	15 (88.2)
Decline to answer	1 (4.3)	0 (0.0)
How much did participation in this study help you improve adherence to your medications? *N* (%)		
A great deal–a lot	10 (43.5)	7 (41.2)
Moderately	5 (21.7)	3 (17.7)
A little–not at all	7 (30.4)	5 (29.4)
Decline to answer	1 (4.3)	2 (11.8)
How much did participation in this study reduce your substance use? *N* (%)		
A great deal–a lot	6 (26.1)	5 (29.4)
Moderately	3 (13.0)	2 (11.8)
A little–not at all	4 (17.4)	8 (47.1)
Decline to answer	10 (43.5)	2 (11.8)
How much did participation in this study help improve your mental health? *N* (%)		
A great deal–a lot	14 (60.6)	15 (88.2)
Moderately	5 (21.7)	2 (11.8)
A little–not at all	2 (8.7)	0 (0.0)
Decline to answer	2 (8.7)	0 (0.0)
How likely would you be to recommend a study like this to a friend? *N* (%)		
Extremely likely–likely	18 (78.3)	17 (100.0)
Somewhat likely–extremely unlikely	4 (17.4)	0 (0.0)
Decline to answer	1 (4.3)	0 (0.0)
In the future, how likely would you be to participate in a similar study where you receive sessions with a counselor over video-counseling? *N* (%)		
Extremely likely–likely	19 (82.6)	17 (100.0)
Somewhat likely–extremely unlikely	2 (8.7)	0 (0.0)
Decline to answer	2 (8.7)	0 (0.0)

^a^ Total reflects those who answered “Yes” to “Did you meet with the counselor in person before beginning your video-counseling sessions?”

^b^ Total reflects those who answered “No” to “Did you meet with the counselor in person before beginning your video-counseling sessions?”

Y2TEC, Youth to Telehealth and Texting for Engagement in Care.

Participants responded to a text message query immediately after each session that they strongly agreed or agreed with this statement: “I felt heard, understood, and respected by the counselor” after 82% of the completed sessions. In addition, participants responded that they strongly agreed or agreed with this statement: “overall, today's session was right for me” after 81% of the completed sessions. Approximately 90% of participants reported that the intervention resulted in a great to moderate amount of improvement in their mental health and 40% reported that the intervention helped a great to moderate amount to reduce their substance use. In addition, 63% of participants reported that the intervention resulted in a great to moderate improvement in their ART adherence.

### HIV treatment engagement

At baseline, all participants reported being on ART, with 90% of the sample reporting an undetectable HIV viral load (Table 3). Despite a mean self-reported ART adherence of ∼89%, 62% of participants reported missing any medication doses over the past 30 days, and 24% reported having good to very poor ART adherence. Very good to excellent ART adherence rating increased in the intervention group (from 76% to 87% to 94% at baseline, 4 months, and 8 months, respectively) and decreased in the waitlist control group (from 89% to 82%). At baseline, 68% of the intervention group had “adequate” HIV knowledge, which increased to 83% after 4 months of the intervention. At 4 months, 55% of the wait-list control group had “adequate” HIV knowledge, which increased to 77% after 4 months of the intervention.

**Table 3.** HIV at Baseline, 4 Months, and 8 Months by Study Arm

	Intervention			Waitlist control		
	Baseline	4 Months	8 Months	Baseline	4 Months	8 Months
	N = 25	N = 23	N = 17	N = 25	N = 20	N = 17
Viral load, *N* (%)						
Detectable	0 (0.0)	0 (0.0)	0 (0.0)	2 (8.0)	1 (5.0)	3 (17.6)
Undetectable	23 (92.0)	22 (95.7)	16 (94.1)	22 (88.0)	18 (90.0)	14 (82.4)
Do not know or decline to answer	2 (8.0)	1 (4.3)	1 (5.9)	1 (4.0)	1 (5.0)	0 (0.0)
On ART, *N* (%)						
Yes	25 (100.0)	23 (100.0)	17 (100.0)	25 (100.0)	19 (95.0)	17 (100.0)
No	0 (0.0)	0 (0.0)	0 (0.0)	0 (0.0)	1 (5.0)	0 (0.0)
Missed doses in the past 30 days,^a^*N* (%)						
None	10 (40.0)	7 (30.4)	8 (47.1)	9 (36.0)	5 (26.3)	5 (29.4)
Any	15 (60.0)	16 (69.6)	9 (52.9)	16 (64.0)	14 (73.7)	12 (70.6)
Take ART correctly in past 30 days,^a^*N* (%)						
Always	13 (52.0)	13 (56.5)	13 (76.5)	11 (44.0)	7 (36.8)	7 (41.2)
Not always	12 (48.0)	10 (43.5)	4 (23.5)	14 (56.0)	12 (63.2)	10 (58.8)
Rate ART ability,^a^*N* (%)						
Very good–excellent	19 (76.0)	20 (87.0)	16 (94.1)	19 (76.0)	17 (89.4)	14 (82.4)
Good–very poor	6 (24.0)	3 (13.0)	1 (5.9)	6 (24.0)	2 (10.6)	3 (17.6)
Self-report medication adherence, mean (SD)	87.4 (15.2)	90.9 (9.9)	91.8 (10.1)	90.3 (7.8)	87.7 (10.5)	87.4 (11.2)
HIV treatment knowledge, *N* (%)						
Adequate	17 (68.0)	19 (82.6)	12 (70.6)	14 (56.0)	11 (55.0)	13 (76.5)
Inadequate	8 (32.0)	4 (17.4)	5 (29.4)	11 (44.0)	9 (45.0)	4 (23.5)

^a^ Total reflects that these questions were only asked of those who answered “Yes” to “Are you on ART?”

ART, antiretroviral therapy; SD, standard deviation.

### Mental health and substance use

Among participants reporting mental health challenges, only 10% noted having ever received mental health services, and among those who reported substance use challenges, only 18.8% reported ever receiving substance use services. Moderate to severe depressive symptoms decreased from among 36% of participants in the intervention arm at baseline to 35% to 24% at 4 and 8 months, respectively. In the waitlist control arm, moderate to severe depressive symptom decreased from 24% at baseline to 15% and 12% at 4 and 8 months, respectively (Table 4). At baseline, 28% of participants in the intervention arm had anxiety scores that warranted further evaluation, which increased to 30% at 4 months and decreased to 6% at 8 months. In the waitlist control arm at 4 months, 20% of participants reported anxiety scores that recommended further evaluation, which decreased to 6% at 8 months. Mean ACES was 7.0 (SD = 2.4), with 86% of participants reporting 5–10 ACES.

**Table 4.** Psychosocial Measures and Substance Use at Baseline, 4 Months, and 8 Months by Study Arm

	Intervention			Waitlist control		
	Baseline	4 Months	8 Months	Baseline	4 Months	8 Months
	N = 25	N = 23	N = 17	N = 25	N = 20	N = 17
PHQ-9, *N* (%)						
None–minimal	11 (44.0)	10 (43.5)	5 (29.4)	12 (48.0)	12 (60.0)	10 (58.8)
Mild	5 (20.0)	5 (21.7)	8 (47.1)	9 (36.0)	5 (25.0)	5 (29.4)
Moderate–severe	9 (36.0)	8 (34.8)	4 (23.5)	4 (24.0)	3 (15.0)	2 (11.8)
PCL, *N* (%)						
PTSD unlikely	18 (72.0)	18 (78.3)	15 (88.2)	23 (92.0)	17 (85.0)	16 (94.1)
PTSD likely	7 (28.0)	5 (21.7)	2 (11.8)	2 (8.0)	3 (15.0)	1 (5.9)
PSQI, *N* (%)						
Good sleep quality	10 (40.0)	9 (39.1)	6 (35.3)	5 (20.0)	6 (30.0)	8 (47.1)
Poor sleep quality	14 (56.0)	12 (52.2)	11 (64.7)	19 (76.0)	13 (65.0)	7 (41.2)
Missing	1 (4.0)	2 (8.7)	0 (0.0)	1 (4.0)	1 (5.0)	2 (11.8)
GAD, *N* (%)						
No further evaluation recommended	18 (72.0)	16 (69.6)	16 (94.1)	19 (76.0)	16 (80.0)	16 (94.1)
Further evaluation recommended	7 (28.0)	7 (30.4)	1 (5.9)	6 (24.0)	4 (20.0)	1 (5.9)
HIV stigma mechanism, mean (SD)	7.6 (4.3)	8.7 (3.5)	8.8 (5.0)	8.5 (3.8)	8.6 (3.5)	7.8 (2.9)
SAMHSA mental health stigma assessment, mean (SD)	9.8 (7.8)	9.0 (7.0)	8.7 (6.9)	8.2 (6.9)	7.2 (6.6)	5.2 (6.2)
PROMIS Item Bank–social Isolation score, mean (SD)	38.2 (15.6)	35.4 (16.0)	38.4 (14.0)	30.2 (10.2)	29.5 (11.4)	26.2 (9.5)
Brief resilience scale, mean (SD)	3.6 (0.9)	3.6 (0.9)	3.6 (0.6)	3.3 (0.7)	3.4 (0.9)	3.3 (0.7)
HCP empowerment, mean (SD)	15.2 (5.2)	17.0 (6.4)	16.8 (5.7)	18.4 (9.1)	20.4 (9.3)	18.2 (2.1)
AUDIT, *N* (%)						
Low risk	18 (72.0)	16 (69.6)	12 (70.6)	16 (64.0)	14 (70.0)	12 (70.6)
Moderate–dependent	7 (28.0)	7 (30.4)	5 (29.4)	9 (36.0)	6 (30.0)	5 (29.4)
DAST, *N* (%)						
No problem–low	19 (76.0)	19 (82.6)	14 (82.4)	16 (64.0)	14 (70.0)	11 (64.7)
Moderate–severe	6 (24.0)	4 (17.4)	3 (17.6)	9 (36.0)	6 (30.0)	6 (35.3)
ASSIST^a^, *N* (%)						
No	23 (92.0)	21 (91.3)	16 (94.1)	20 (80.0)	15 (75.0)	15 (88.2)
Yes	2 (8.0)	2 (8.7)	1 (5.9)	5 (20.0)	5 (25.0)	2 (11.8)
SAMHSA alcohol stigma assessment, mean (SD)	11.1 (8.2)	9.9 (6.8)	8.8 (7.1)	8.7 (8.2)	7.7 (6.5)	7.9 (7.1)
SAMHSA drug use stigma assessment, mean (SD)	12.7 (8.7)	12.1 (7.4)	12.0 (8.9)	11.6 (8.7)	8.3 (7.2)	9.5 (7.7)

^a^ Monthly or more frequent drug use (besides marijuana or tobacco), or daily use of marijuana or tobacco.

ASSIST, Alcohol, Smoking, and Substance Involvement Screening Test; AUDIT, Alcohol Use Disorders Identification Test; DAST, Drug Abuse Screening Test; GAD, Generalized Anxiety Disorder; HCP, health care provider; PCL-5, Post-Traumatic Stress Disorder Checklist; PHQ-9, Patient Health Questionnaire; PROMIS, Patient-Reported Outcomes Measurement Information System; PSQI, Pittsburgh Sleep Quality Index; SAMHSA, Substance Abuse and Mental Health Services Administration.

In the intervention arm, 28%, 30%, and 29% of participants reported moderate to dependent alcohol use at baseline, 4 months, and 8 months, respectively. In the waitlist control arm, 36%, 30%, and 30% reported moderate to dependent alcohol use at baseline, 4 months, and 8 months, respectively. Moderate to severe DAST scores changed from 24% to 17% to 18% in the intervention arm at baseline, 4 months, and 8 months, respectively, and 36% to 30% to 35% in the waitlist control arm at baseline, 4 months, and 8 months, respectively. There were slight decreases in SAMHSA mental health, alcohol, and drug use stigma assessments, as well as social isolation score (Table 4).

## Discussion

The Y2TEC pilot study was highly acceptable and feasible among YLWH in the San Francisco Bay Area. We noted improvements in HIV clinical outcomes, mental health, and substance use during the study. Despite high prevalence of history of homelessness and financial hardship, the vast majority (98%) found the video-counseling intervention acceptable. Participants in both arms reported high levels of comfort with a counselor who they had only met once in person or not met in person at all. Given that this was a pilot study with a small sample size, following best practices articulated in the research methods literature, we are not reporting tests of statistical significance and efficacy of the intervention.^41,43,44^

In this pilot study, we were able to meet all predetermined benchmarks for feasibility and acceptability^13^ except recruitment and number of call disconnections. We believe that this was likely due to several reasons. In terms of recruitment challenges, social media platforms used for study advertisement modified their requirements based on European Union's General Data Protection Regulation, which made it substantially more difficult to reach our population based on age. Funding cuts or completion of various research projects at some clinics resulted in cancellation of some programs for YLWH and a reduction in patient engagement at some recruitment sites. Given that the enrollment visit was in -person, many interested individuals found it difficult to schedule time given full-time work and school. In addition, we had slightly more call disconnections than the prespecified benchmark. Based on these findings, for future trials involving YLWH, we will offer enrollment visits online to create a fully virtual/remote care experience, increase the geographic area of recruitment, and partner with social media platforms that are able to target users based on age. We have also developed videoconferencing best practices^45^ to minimize call disconnection in future research.

Mental health care engagement is associated with increased retention in HIV care,^46,47^ higher ART adherence and virological suppression,^48^ and decreased mortality.^47,49^ Approximately one in five adults in the United States experience mental health challenges in a given year; however, only 41% of them will receive mental health services.^50^ In addition, the current social and political moment presents both new challenges and opportunities for managing mental health for YLWH. Given the need for social isolation and fear of SARS-CoV-2, there is a worry that depressive symptoms and mental health challenges may increase, especially in vulnerable populations.^51,52^

Finally, many people in need of mental health and substance use services are not identified as needing these services,^53–58^ in part, due to lack of screening, human resource shortages,^59–61^ fragmented and underfunded service delivery models,^62,63^ and lack of youth-friendly services.^64^ This issue is especially relevant in California where only ∼2% of young adults are engaged with community mental health programs, lower than the nationwide average.^65^

For PLWH and in need of mental health and substance use services, access is complicated by the diversity of insurance and public coverage sources that pay for care. Given the current SARS-CoV-2 pandemic, the use of video-counseling and provision of remote research and services have been increasing^66^; therefore, we believe that there will be increased coverage for service that can help identify and link patients to mental health and substance use services in a remote manner.

Given the substantial challenges to the provision and scale-up of mental health screening and treatment to larger number of individuals, technology-based methods have been offered as a possible approach to efficient and effective mental health care delivery.^67^ Task shifting of aspects of mental health care can expand the workforce's capacity by shifting responsibilities, for example, from psychologists to social workers.^68^ Technology-based approaches can improve scale-up of mental health care and increase support and reduce burden to counselors.^14^ Telephone-, computer-, and internet-based mental health interventions can enhance access^14,69,70^ among YLWH. In Y2TEC, we used technology to allow for remote assessment and brief intervention, which can allow for a more sustainable and efficient mechanism to increase linkage for more intensive traditional mental health and substance use treatment for those who require further care without overburdening mental health providers.

This pilot study was limited due to the recruitment of predominately gay cisgender men in the San Francisco Bay Area. Therefore, results may not be generalizable to other populations. In addition, we had a small sample size and focused on self-reported data. Finally, the Y2TEC intervention requires access to a smartphone or computer to participate in video-counseling sessions; therefore, it may not be applicable to those who do not have access to these tools. However, YLWH represent a population disparately impacted by the HIV epidemic and in need of tailored interventions to meet their specific needs.

Although mental health, substance use, and other psychosocial factors have been shown to contribute to poor HIV treatment adherence among this underserved population, no other known interventions exist that address these factors while taking into consideration the unique culture of YLWH related to technology use. Therefore, to our knowledge, Y2TEC may be the first intervention targeting mental health, substance use, and engagement in HIV care that is specifically focused on and tailored for YLWH. In future studies, we aim to examine the efficacy of this intervention among a larger sample of YLWH, taking into account the lessons learned in this pilot study.

## Abbreviations Used

ACES: Adverse Childhood Experiences Score
ART: antiretroviral therapy
ASSIST: Alcohol, Smoking, and Substance Involvement Screening Test
AUDIT: Alcohol Use Disorders Identification Test
DAST: Drug Abuse Screening Test
GAD: Generalized Anxiety Disorder
PCL-5: PTSD Checklist
PHQ-9: Patient Health Questionnaire
PROMIS: Patient-Reported Outcomes Measurement Information System
PSQI: Pittsburgh Sleep Quality Index
PTSD: Post-Traumatic Stress Disorder
SAMHSA: Substance Abuse and Mental Health Services Administration
SARS-CoV-2: severe acute respiratory syndrome coronavirus 2
SD: standard deviation
Y2TEC: Youth to Telehealth and Texting for Engagement in Care
YLWH: youth and young adults living with HIV

## Notes

^*^ McCuistian C, Wootton AR, Legnitto DA, et al. Development of a videocounseling intervention to address HIV care, mental health, and substance use challenges for young adults. BMJ Open (Under review).
